# Soluble programmed cell death-1 predicts hepatocellular carcinoma development during nucleoside analogue treatment

**DOI:** 10.1038/s41598-021-03706-w

**Published:** 2022-01-07

**Authors:** Ritsuzo Kozuka, Masaru Enomoto, Minh Phuong Dong, Hoang Hai, Le Thi Thanh Thuy, Naoshi Odagiri, Kanako Yoshida, Kohei Kotani, Hiroyuki Motoyama, Etsushi Kawamura, Atsushi Hagihara, Hideki Fujii, Sawako Uchida-Kobayashi, Akihiro Tamori, Norifumi Kawada

**Affiliations:** Department of Hepatology, Graduate School of Medicine, Osaka City University, 1-4-3 Asahimachi, Abeno-ku, Osaka, 545-8585 Japan

**Keywords:** Gastroenterology, Hepatology, Hepatitis, Viral hepatitis

## Abstract

Soluble immune checkpoint molecules are emerging novel mediators of immune regulation. However, it is unclear whether soluble immune checkpoint proteins affect the development of hepatocellular carcinoma (HCC) during nucleos(t)ide analogue (NA) treatment in patients with chronic hepatitis B virus infection. This study included 122 NA-naïve patients who received NA therapy. We assessed the associations of clinical factors, including soluble immune checkpoint proteins, with HCC development during NA treatment. The baseline serum concentrations of 16 soluble immune checkpoint proteins were measured using multiplexed fluorescent bead-based immunoassay. In total, 13 patients developed HCC during the follow-up period (median duration, 4.3 years). Of the 16 proteins, soluble inducible T-cell co-stimulator (≥ 164.71 pg/mL; *p* = 0.014), soluble programmed cell death-1 (sPD-1) (≤ 447.27 pg/mL; *p* = 0.031), soluble CD40 (≤ 493.68 pg/mL; *p* = 0.032), and soluble herpes virus entry mediator (≤ 2470.83 pg/mL; *p* = 0.038) were significantly associated with HCC development (log-rank test). In multivariate analysis, an sPD-1 level ≤ 447.27 pg/mL (*p* = 0.014; hazard ratio [HR], 4.537) and α-fetoprotein level ≥ 6.4 ng/mL (*p* = 0.040; HR, 5.524) were independently and significantly associated with HCC development. Pre-treatment sPD-1 is a novel predictive biomarker for HCC development during NA treatment.

## Introduction

Hepatitis B virus (HBV) infection is an important public health issue and a leading cause of cirrhosis and hepatocellular carcinoma (HCC), being responsible for more than 686,000 deaths annually^1^.

Nucleos(t)ide analogues (NAs) such as entecavir and tenofovir are widely used in patients with chronic HBV infection. Moreover, NA treatment for chronic HBV infection has been reported to not only suppress HBV replication but also reduce the risk of HCC development2–4. However, some patients still develop HCC despite effective treatment with NA, especially when HCC-related risk factors are reported during the natural course3–7. We reported that human leukocyte antigen -DQA1/DRB1 polymorphism and cirrhosis were risk factors for HCC development during entecavir treatment8. Therefore, understanding the HCC-risked factors before the start of NA treatment may improve HCC surveillance in patients with chronic HBV infection.

The risk factors for developing HCC can be classified as host, viral, and environmental factors. In particular, it is important to investigate host immunological factors for HCC development because immune dysregulation with the exhaustion of HBV-specific CD8+ T cells plays an important role in persistent HBV infection and contributes to the immunopathogenesis of HBV-associated liver diseases, including hepatocarcinogenesis^9–17^.

Circulating soluble immune checkpoint proteins, members of a family of full-length receptors produced via mRNA expression or the cleavage of membrane-bound proteins, have been studied as host immunological factors in various cancers^18^. Moreover, these soluble immune checkpoint proteins can diffuse in the serum. However, the functions of these proteins are unclear; soluble forms of inhibitory factors are not necessarily involved in negative immune regulation, and vice versa. Soluble immune checkpoint proteins, such as soluble B- and T-lymphocyte attenuator (sBTLA)^19^, soluble T-cell immunoglobulin and mucin domain-3 (sTIM-3)^20^, soluble herpes virus entry mediator (sHVEM)^21^, soluble programmed cell death-1 (sPD-1)^22–24^, soluble cytotoxic T-lymphocyte associated antigen 4 (sCTLA-4)^25^, and soluble programmed cell death-ligand 1 (sPD-L1)^24,26,27^, are linked to the development and prognosis of HCC, and are potential biomarkers and therapeutic targets.

However, it is unclear whether soluble immune checkpoint proteins affect HCC development during NA treatment in patients with chronic HBV infection. Therefore, we evaluated host immunological factors associated with HCC development, *i*.*e*., soluble immune checkpoint proteins, during NA treatment.

## Results

### Baseline characteristics

Table 1 summarizes the baseline characteristics of the patients. The median (range) age was 45 (23–79) years, and there were 78 (63.9%) males and 22 (18.0%) patients with cirrhosis. A total of 104 (85.2%) patients had HBV genotype C. Although the baseline characteristics were similar to those reported in our previous study^8^, the median follow-up duration was extended from 4.4 (range, 1.0–10.7) to 6.2 (range, 1.1–13.3) years.

**Table 1:** Baseline characteristics of the patients.

	Total
	(n = 122)
**Clinical factor**	
Age (years)	45 (23–79)
Sex (male)	78 (63.9%)
Body mass index (kg/m^2^)	22.3 (14.7–38.9)
Cirrhosis	22 (18.0%)
HCC family history ( +)	11 (9.0%)
Previous interferon treatment ( +)	25 (20.5%)
Alcohol consumption ( +)	17 (13.9%)
Cigarette smoking ( +)	41 (33.6%)
Diabetes mellitus ( +)	7 (5.7%)
Fatty liver ( +)	36 (29.5%)
Platelet count (×10^3^/mm^3^)	165 (50–403)
Aspartate aminotransferase (U/L)	72 (22–872)
Alanine aminotransferase (U/L)	113 (16–2605)
γ-glutamyltransferase (U/L)	52 (10–474)
Total bilirubin (mg/dL)	0.8 (0.2–31.1)
Albumin (g/dL)	4.1 (2.1–5.0)
Prothrombin time (%)	88 (33–134)
α-fetoprotein (ng/mL)	5.8 (< 2.0–903.8)
HBV genotype (A/B/C/D/N.D.)	3/12/104/1/2
HBeAg positivity	59 (48.4%)
HBsAg (log IU/mL)	3.53 (-0.85–5.35)
HBV-DNA (log copies/mL)	7.3 (3.1– > 9.1)
Precore G1896A	39 (32.0%)
Basic core promoter A1762T/G1764A	85 (69.7%)
Hyaluronic acid (ng/mL)	71.7 (< 10–1480)
Type III procollagen-N-peptide (U/mL)	0.9 (0.4–3.9)
Type IV collagen (ng/mL)	179 (88–695)
M2BPGi (C.O.I)	1.25 (0.24–12.29)
AST to platelet ratio index	1.45 (0.26–26.16)
Fibrosis-4 index	2.11 (0.54–12.16)
Treatment duration (years)	6.2 (1.1–13.3)
**Soluble immune checkpoint proteins**	
sBTLA (pg/mL)	92.36 (4.88–7379.58)
sCD27 (pg/mL)	2240.59 (275.77–90,205.76)
sCD28 (pg/mL)	1829.45 (452.34–40,812.96)
sTIM-3 (pg/mL)	3182.68 (22.39–26,629.55)
sHVEM (pg/mL)	2806.94 (514.79–34,673.87)
sCD40 (pg/mL)	482.43 (217.88–14,447.65)
sGITR (pg/mL)	0.49 (0.49–1195.54)
sLAG-3 (pg/mL)	10,062.07 (870.17–101,198.27)
sTLR-2 (pg/mL)	337.92 (131.75–8434.07)
sGITRL (pg/mL)	26.67 (2.441–1233.55)
sPD-1 (pg/mL)	523.72 (131.95–6310.53)
sCTLA-4 (pg/mL)	14.46 (2.84–301.54)
sCD80 (pg/mL)	12.18 (1.22–4117.63)
sCD86 (pg/mL)	334.07 (56.92–6589.98)
sPD-L1 (pg/mL)	23.23 (1.64–550.01)
sICOS (pg/mL)	89.96 (4.88–3717.92)

The values are medians (with ranges) or numbers (with percentages).

HBV, hepatitis B virus; N.D., not determined; HBeAg, hepatitis B e antigen; HBsAg, hepatitis B surface antigen; sBTLA, soluble B- and T-lymphocyte attenuator; sCD27, soluble CD27; sCD28, soluble CD28; sTIM-3, soluble T-cell immunoglobulin and mucin domain-3; sHVEM, soluble herpes virus entry mediator; sCD40, soluble CD40; sGITR, soluble glucocorticoid-induced TNFR-related; sLAG-3, soluble lymphocyte-activation gene 3; sTLR-2, soluble toll-like receptor 2; sGITRL, soluble glucocorticoid-induced TNFR-related ligand; sPD-1, soluble programmed cell death-1; sCTLA-4, soluble cytotoxic T-lymphocyte associated antigen 4; sCD80, soluble CD80; sCD86, soluble CD86; sPD-L1, soluble programmed cell death-ligand 1; sICOS, soluble inducible T-cell co-stimulator.

### Cumulative rates of HCC development according to clinical factors at baseline

In our previous report^8^, 10 patients developed HCC during the follow-up period (median duration, 3.3 [range, 1.1–6.9] years); since then, an additional 3 patients have developed HCC (median duration, 4.3 [range, 1.1–7.6] years) (Table 2). In accordance with our previous report^8^, liver fibrosis, the platelet count, the fibrosis-4 (FIB-4) index, age, type IV collagen, and α-fetoprotein (AFP) were significantly associated with HCC development according to the log-rank test. Moreover, hyaluronic acid and hepatitis B surface antigen (HBsAg) were associated with HCC development (Supplementary Fig. 1).

**Table 2:**
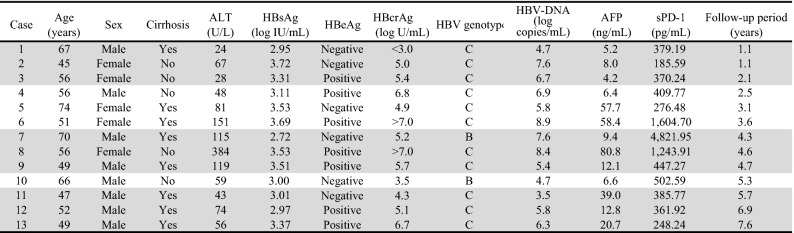
Profiles of patients who developed HCC during entecavir treatment.

The patients whose data are shaded gave serum samples 6 and 12 months after commencement of entecavir treatment, 6 and 12 months before HCC development, and at the time of HCC development.

HCC, hepatocellular carcinoma; ALT, alanine aminotransferase; HBsAg, hepatitis B surface antigen; HBeAg, hepatitis B e antigen; HBcrAg, hepatitis B core-related antigen; HBV, hepatitis B virus; AFP, α-fetoprotein; sPD-1, soluble programmed cell death-1.

### Cumulative rates of HCC development according to soluble immune checkpoint protein levels at baseline

**Figure 1:**
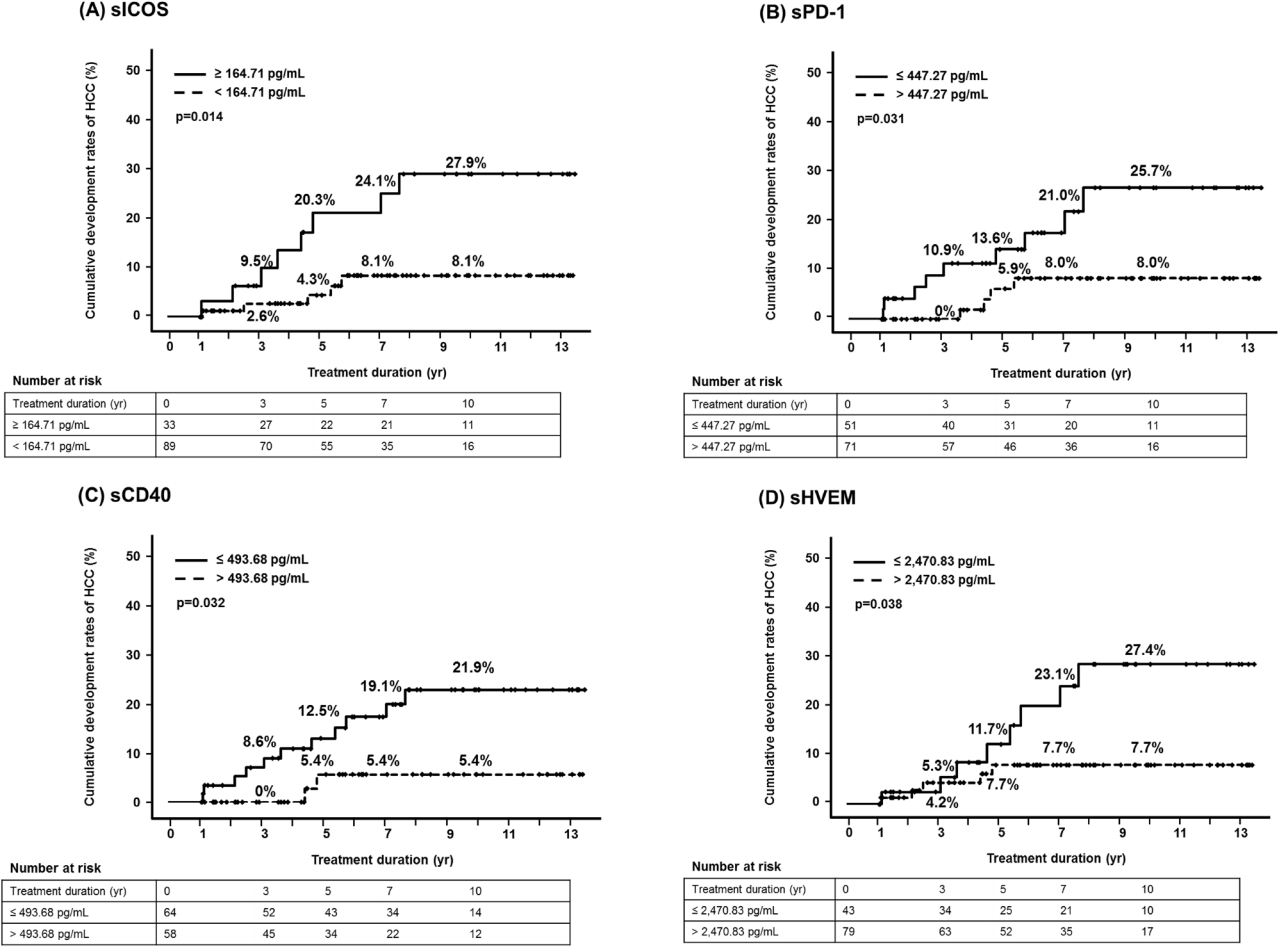
Cumulative rates of hepatocellular carcinoma (HCC) according to (**A**) soluble inducible T-cell co-stimulator (sICOS), (**B**) soluble programmed cell death-1 (sPD-1), (**C**) soluble CD40 (sCD40), and (**D**) soluble herpes virus entry mediator (sHVEM) levels at baseline.

The cumulative rates of HCC development according to the serum soluble inducible T-cell co-stimulator (sICOS) level are shown in Fig. 1A. The cumulative rates of HCC development at 3, 5, 7, and 10 years were 9.5%, 20.3%, 24.1%, and 27.9%, respectively, in patients with sICOS ≥ 164.71 pg/mL (*n* = 33), and 2.6%, 4.3%, 8.1%, and 8.1%, respectively, in those with sICOS < 164.71 pg/mL (*n* = 89) (*p* = 0.014).

The cumulative rates of HCC development according to the serum sPD-1 level are shown in Fig. 1B. The cumulative rates of HCC development at 3, 5, 7, and 10 years were 10.9%, 13.6%, 21.0%, and 25.7%, respectively, in patients with sPD-1 ≤ 447.27 pg/mL (*n* = 51), and 0%, 5.9%, 8.0%, and 8.0%, respectively, in those with sPD-1 > 447.27 pg/mL (*n* = 71) (*p* = 0.031).

The cumulative rates of HCC development according to the serum soluble (s) CD40 level are shown in Fig. 1C. The cumulative rates of HCC development at 3, 5, 7, and 10 years were 8.6%, 12.5%, 19.1%, and 21.9%, respectively, in patients with sCD40 ≤ 493.68 pg/mL (*n* = 64), and 0%, 5.4%, 5.4%, and 5.4%, respectively, in those with sCD40 > 493.68 pg/mL (*n* = 58) (*p* = 0.032).

The cumulative rates of HCC development according to the serum sHVEM level are shown in Fig. 1D. The cumulative rates of HCC development at 3, 5, 7, and 10 years were 5.3%, 11.7%, 23.1%, and 27.4%, respectively, in patients with sHVEM ≤ 2470.83 pg/mL (*n* = 43), and 4.2%, 7.7%, 7.7%, and 7.7%, respectively, in those with sHVEM > 2470.83 pg/mL (*n* = 79) (*p* = 0.038).

There were no significant associations between HCC development and the levels of soluble immune checkpoint proteins (sBTLA, sCD27, sCD28, sTIM-3, soluble glucocorticoid-induced TNFR-related [sGITR], soluble lymphocyte-activation gene 3 [sLAG-3], soluble toll-like receptor 2 [sTLR-2], soluble glucocorticoid-induced TNFR-related ligand [sGITRL], sCTLA-4, sCD80, sCD86, and sPD-L1) (Supplementary Fig. 2).

### Predictive factors for HCC development during entecavir treatment

As we reported previously^8^, the FIB-4 index, platelet count, cirrhosis status, age, and AFP were associated with HCC development during entecavir treatment in univariate analyses. In addition, levels of type IV collagen ≥ 200 ng/mL (*p* = 0.011; hazard ratio [HR], 5.300; 95% confidence interval [CI], 1.458–19.270), hyaluronic acid ≥ 143 ng/mL (*p* = 0.017; HR, 3.922; 95% CI 1.281–12.008), HBsAg ≤ 3.53 log IU/mL (*p* = 0.027; HR, 5.507; 95% CI 1.220–24.859), sICOS ≥ 164.71 pg/mL (*p* = 0.022; HR, 3.713; 95% CI 1.210–11.393), sPD-1 ≤ 447.27 pg/mL (*p* = 0.042; HR, 3.393; 95% CI 1.044–11.033), and sHVEM ≤ 2470.83 pg/mL (*p* = 0.049; HR, 3.078; 95% CI 1.006–9.416) were significantly associated with HCC development during entecavir treatment (Table 3).

**Table 3:** Predictive factors of HCC development during entecavir treatment.

Factor	Category	Univariate analysis				Multivariate analysis			
		HR	95% CI		*p*-value	HR	95% CI		*p*-value
**Clinical factor**									
Age (years)	≥ 49	7.458	1.652	33.669	0.0090*				
Sex	Male	0.839	0.274	2.569	0.76				
Body mass index (kg/m^2^)	≥ 21.9	2.665	0.733	9.691	0.14				
Cirrhosis	(+)	8.008	2.611	24.562	0.0003*				
HCC family history	(+)	1.979	0.438	8.936	0.38				
Previous interferon treatment	(+)	1.128	0.310	4.103	0.85				
Alcohol consumption	(+)	1.621	0.445	5.902	0.46				
Cigarette smoking	(+)	1.154	0.377	3.530	0.80				
Diabetes mellitus	(+)	2.836	0.627	12.828	0.18				
Fatty liver	(+)	0.196	0.025	1.506	0.12				
Platelet count (×10^3^/mm^3^)	≤ 117	9.546	2.928	31.127	0.0002*				
Aspartate aminotransferase (U/L)	≤ 80	1.517	0.467	4.931	0.49				
Alanine aminotransferase (U/L)	≤ 81	3.058	0.941	9.936	0.063				
γ-glutamyltransferase (U/L)	≥ 40	2.082	0.573	7.565	0.27				
Total bilirubin (mg/dL)	≥ 0.9	2.268	0.742	6.934	0.15				
Albumin (g/dL)	≤ 4.1	2.847	0.783	10.352	0.11				
Prothrombin time (%)	≤ 84	2.535	0.740	8.680	0.14				
α-fetoprotein (ng/mL)	≥ 6.4	5.812	1.288	26.231	0.022*	5.524	1.084	28.164	0.040*
HBV genotype	C	0.847	0.187	3.837	0.83				
HBeAg	Positivity	1.491	0.500	4.447	0.47				
HBsAg (log IU/mL)	≤ 3.53	5.507	1.220	24.859	0.027*				
HBV-DNA (log copies/mL)	≤ 6.9	2.264	0.696	7.362	0.17				
Precore	G1896A	1.230	0.402	3.764	0.72				
Basic core promoter	A1762T/G1764A	1.070	0.289	3.962	0.92				
Hyaluronic acid (ng/mL)	≥ 143	3.922	1.281	12.008	0.017*				
Type III procollagen-N-peptide (U/mL)	≥ 0.9	1.770	0.579	5.414	0.32				
Type IV collagen (ng/mL)	≥ 200	5.300	1.458	19.270	0.011*				
M2BPGi (C.O.I)	≥ 1.71	2.286	0.767	6.811	0.14				
AST to platelet ratio index	≥ 1.58	3.108	0.954	10.127	0.060				
Fibrosis-4 index	≥ 4.08	10.117	3.093	33.089	0.0001*				
**Soluble immune checkpoint proteins**									
sBTLA (pg/mL)	≥ 100.68	2.093	0.641	6.831	0.22				
sCD27 (pg/mL)	≤ 2262.18	2.175	0.668	7.079	0.20				
sCD28 (pg/mL)	≥ 1705.07	2.584	0.711	9.393	0.15				
sTIM-3 (pg/mL)	≤ 3184.99	1.861	0.573	6.048	0.30				
sHVEM (pg/mL)	≤ 2470.83	3.078	1.006	9.416	0.049*				
sCD40 (pg/mL)	≤ 493.68	4.518	1.000	20.405	0.050				
sGITR (pg/mL)	≤ 0.49	2.984	0.661	13.476	0.16				
sLAG-3 (pg/mL)	≤ 11,206.70	1.704	0.524	5.539	0.38				
sTLR-2 (pg/mL)	≤ 321.19	2.554	0.786	8.301	0.12				
sGITRL (pg/mL)	≤ 26.52	2.429	0.747	7.892	0.14				
sPD-1 (pg/mL)	≤ 447.27	3.393	1.044	11.033	0.042*	4.537	1.363	15.103	0.014*
sCTLA-4 (pg/mL)	≥ 12.63	3.461	0.766	15.631	0.11				
sCD80 (pg/mL)	≥ 12.99	1.752	0.573	5.359	0.33				
sCD86 (pg/mL)	≥ 310.61	2.511	0.690	9.143	0.16				
sPD-L1 (pg/mL)	≤ 24.67	2.435	0.670	8.856	0.18				
sICOS (pg/mL)	≥ 164.71	3.713	1.210	11.393	0.022*				

*p < 0.05. HCC, hepatocellular carcinoma.

HBV, hepatitis B virus; HBeAg, hepatitis B e antigen; HBsAg, hepatitis B surface antigen; sBTLA, soluble B- and T-lymphocyte attenuator; sCD27, soluble CD27; sCD28, soluble CD28; sTIM-3, soluble T-cell immunoglobulin and mucin domain-3; sHVEM, soluble herpes virus entry mediator; sCD40, soluble CD40; sGITR, soluble glucocorticoid-induced TNFR-related; sLAG-3, soluble lymphocyte-activation gene 3; sTLR-2, soluble toll-like receptor 2; sGITRL, soluble glucocorticoid-induced TNFR-related ligand; sPD-1, soluble programmed cell death-1; sCTLA-4, soluble cytotoxic T-lymphocyte-associated antigen 4; sCD80, soluble CD80; sCD86, soluble CD86; sPD-L1, soluble programmed cell death-ligand 1; sICOS, soluble inducible T-cell co-stimulator.

From multivariate analysis, a serum sPD-1 level ≤ 447.27 pg/mL (*p* = 0.014; HR, 4.537; 95% CI 1.363–15.103) and AFP level ≥ 6.4 ng/mL (*p* = 0.040; HR, 5.524; 95% CI 1.084–28.164) were independently and significantly associated with HCC development during entecavir treatment (Table 3).

### Cumulative rates of HCC development according to the combination of serum pre-treatment sPD-1 and AFP levels

**Figure 2:**
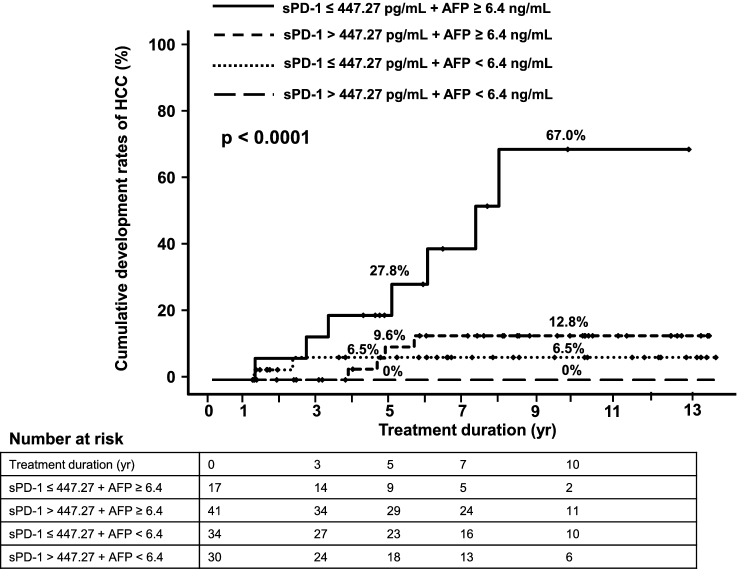
Cumulative rates of HCC development according to the combination of serum pre-treatment sPD-1 and α-fetoprotein (AFP) levels.

When classified according to serum pre-treatment sPD-1 and AFP levels, the 10-year cumulative rates of HCC development were 0%, 6.5%, 12.8%, and 67.0% in the sPD-1 > 447.27 pg/mL + AFP < 6.4 ng/mL group (*n* = 30), sPD-1 ≤ 447.27 pg/mL + AFP < 6.4 ng/mL group (*n* = 34), sPD-1 > 447.27 pg/mL + AFP ≥ 6.4 ng/mL group (*n* = 41), and sPD-1 ≤ 447.27 pg/mL + AFP ≥ 6.4 ng/mL group (*n* = 17), respectively (*p* < 0.0001) (Fig. 2).

### Relationships between the serum sPD-1 level and clinical/virological characteristics

**Figure 3:**
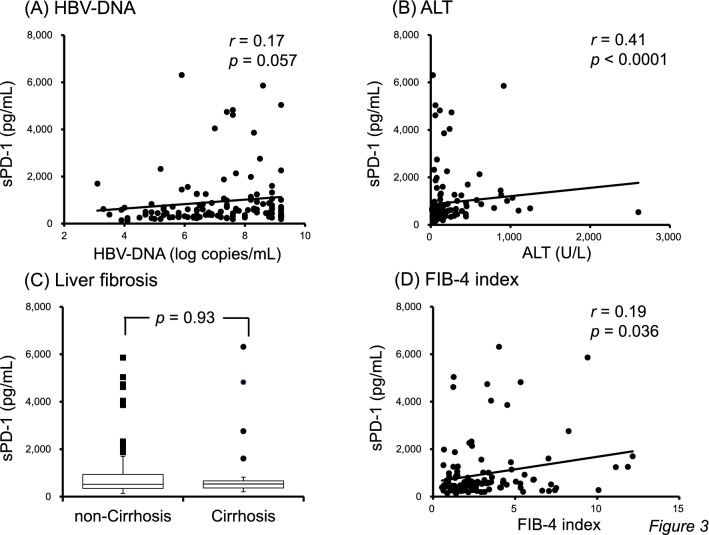
Correlation between the serum sPD-1 level and (**A**) hepatitis B virus (HBV)-DNA and (**B**) alanine aminotransferase (ALT) levels. Serum sPD-1 levels in non-cirrhotic and cirrhotic patients (**C**). Correlation between the serum sPD-1 level and fibrosis-4 (FIB-4) index (**D**).

The serum sPD-1 level at baseline was not strongly correlated with the HBV-DNA level (*p* = 0.057, *r* = 0.17; Fig. 3A), but was positively correlated with the alanine aminotransferase (ALT) level (*p* < 0.0001, *r* = 0.41; Fig. 3B). In addition, the baseline sPD-1 level was similar between non-cirrhotic and cirrhotic patients (median 519.35 pg/mL vs. 534.62 pg/mL, *p* = 0.93; Fig. 3C), and the level did not correlate strongly with the FIB-4 index (*p* = 0.036, *r* = 0.19; Fig. 3D).

### Changes in serum sPD-1 level in patients who developed HCC during entecavir treatment

**Figure 4:**
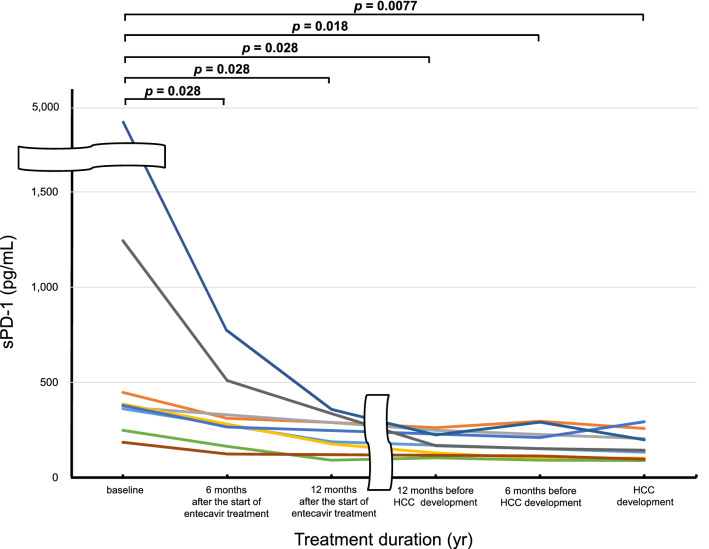
Dynamic changes in the serum sPD-1 level in patients who developed HCC during entecavir treatment.

We analyzed the changes in serum sPD-1 level in 9 (cases 1, 2, 3, 7, 8, 9, 11, 12, and 13; Table 2) of 13 patients who developed HCC during entecavir treatment (Fig. 4). The serum sPD-1 level decreased rapidly after 6 months of entecavir treatment. Significant decreases were found at 6 (*p* = 0.028) and 12 (*p* = 0.028) months after the start of entecavir treatment, at 12 (*p* = 0.028) and 6 (*p* = 0.018) months before HCC development, and at the time of HCC development (*p* = 0.0077), compared with baseline values. Moreover, no re-increase in the serum sPD-1 level at HCC development was observed.

## Discussion

To our knowledge, this is the first evaluation of the associations between soluble immune checkpoint proteins and HCC development during NA treatment in patients with chronic HBV infection. Our results indicate that pre-treatment sPD-1 and AFP are predictive of HCC development during NA treatment, and the predictive accuracy is increased using a combination of serum pre-treatment sPD-1 and AFP levels (Fig. 2).

Soluble immune checkpoint proteins, such as sBTLA, sTIM-3, sHVEM, sPD-1, sCTLA-4, and sPD-L1, may be predictive of the treatment response and prognosis in patients with HCC^19–27^. However, the associations between these proteins and HCC development during NA treatment were unknown. In this study, we measured the concentrations of 16 soluble immune checkpoint proteins in the serum of patients treated with NAs, and detected a significant association between the serum pre-treatment sPD-1 level and HCC development during NA treatment (Table 3).

T cells play an important role in chronic HBV infection because persistent exposure to high concentrations of viral antigens leads to various degrees of T-cell functional impairment, termed T-cell exhaustion^17,23^. PD-1 is expressed on the surface of activated T cells^28^ and has two ligands, PD-L1 and PD-L2^29^. The binding of PD-1 to its ligands may inhibit activated T cells and plays a critical role in T-cell exhaustion^30^. Therefore, PD-1 expression is correlated with the exhaustion of T cells in chronic infections and cancers^31^. Soluble forms of PD-1 have been detected in blood. Unlike PD-1, the function of sPD-1 is unclear. However, sPD-1 can compete with PD-1 by binding to PD-L1 and blocking PD-1/PD-L1 interactions, enhancing antigen-specific T-cell immunity and dendritic cell maturation^32^, restore the proliferative response of virus-specific CD4 and CD8 T cells during chronic infection^33,34^, and enhance antitumor responses^35^.

Previous studies have reported relationships between the serum sPD-1 level and clinical/virological characteristics in patients with chronic HBV infection^22,36,37^. According to these reports, the serum sPD-1 level is significantly positively correlated with the HBV-DNA and ALT levels and similar between cases of non-cirrhosis and cirrhosis, largely consistent with our findings (Fig. 3). The serum sPD-1 level in patients with non-cirrhosis, cirrhosis, and HCC was significantly higher than that in asymptomatic carriers. Patients with chronic HBV infection and HCC have the highest sPD-1 level. Xia et al. reported that the serum sPD-1 level decreased gradually after NA treatment in most cases^36^, consistent with our results. We speculate that the decrease in the sPD-1 level during NA treatment was associated with suppression of HBV DNA replication and improvement of liver inflammation. Interestingly, the serum sPD-1 level did not increase again upon HCC development during NA treatment (Fig. 4), although increased sPD-1 reflects an immune response to HBV infection or HCC. These findings suggest that NA treatment reduces the immune response to HBV infection and HCC by suppressing HBV replication and HCC development, and on-treatment sPD-1 is not useful as a biomarker for identifying HCC during NA treatment.

Chang et al. performed a retrospective study of 120 patients with HCC receiving radical resection and showed that a low level of sPD-1 at baseline was correlated with poor overall survival as well as a trend toward shortened disease-free survival in patients with HBV-related HCC^24^. These findings suggest that a low pre-treatment sPD-1 level worsens the prognosis of HCC. This is consistent with our result that a lower pre-treatment sPD-1 level was a risk factor for HCC development during NA treatment in patients with chronic HBV infection.

Cheng et al. showed that a high sPD-1 level (> 282 pg/mL) at baseline was associated with a twofold increase in the risk of HCC in a cohort of HBV patients^23^. By contrast, our findings showed that patients with chronic HBV infection with a lower sPD-1 level (≤ 447.27 pg/mL) at baseline had a higher risk of developing HCC during NA treatment. This discrepancy could be attributed to the difference in the background of the study population between Cheng et al*.*’s and our studies. The study population in Cheng et al*.* consisted of asymptomatic carriers not on NA treatment; the serum sPD-1 level in asymptomatic carriers is significantly lower than in patients with chronic HBV infection^22^. In our study, serum pre-treatment sPD-1 was detected only in the analysis limited to patients treated with NAs; NAs are administered to a large number of patients with chronic HBV infection, who may have a higher risk of HCC. In addition, we speculate that the impact of competition with PD-1 for binding to PD-L1 and blocking PD-1/PD-L1 interactions was reduced in patients with chronic HBV infection with a lower pre-treatment sPD-1 level, leading to a reduced antitumor response. This is consistent with a previous report^35^. Hence, our findings imply that patients with a lower sPD-1 level before the initiation of NA treatment should be followed up carefully as a population at higher risk of HCC, despite a report that NA treatment for chronic HBV infection reduces the risk of HCC development^2–4^.

Previous reports indicated that the levels of the hepatitis B core-related antigen (HBcrAg) usefully predicted HCC development; patients with higher pre-treatment HBcrAg levels were at greater risk of HCC development during NA treatment^38–41^. No report has yet evaluated the associations between HBcrAg and sPD-1 levels for HCC development during NA treatment. We found that patients with a lower baseline sPD-1 level were at higher risk of HCC development during NA treatment, but the pre-treatment HBcrAg level was not always elevated in such patients (Table 2).

Our study had some limitations. First, it was a single-center study conducted on a relatively small scale. Second, most of the study population consisted of patients with HBV genotype C, and it is unclear whether the results can be extended to patients with different HBV genotypes because HBV genotype C is associated with a higher risk of HCC development. Therefore, further large-scale studies with patients with various HBV genotypes are necessary.

In conclusion, our findings suggest that pre-treatment sPD-1 is a novel predictive biomarker for HCC development during NA treatment.

## Patients and methods

### Patients

Of 127 patients in our previous study^8^, 122 patients for whom stored serum samples collected before the start of entecavir treatment were available were included in this retrospective study. These 122 NA-naïve patients with chronic HBV infection, verified as HBsAg-positive and HBV DNA-positive for at least 6 months before treatment, underwent entecavir therapy from September 2006 to September 2016 at Osaka City University Hospital. The inclusion criteria were as follows: persistent elevation of the serum ALT level (≥ 31 U/L) and the HBV DNA level (≥ 4.0 log copies/mL; equivalent to 3.3 log IU/mL) as well as the presence of advanced fibrosis even if the ALT level was within the normal range according to published guidelines^1,42,43^, no clinical signs of HCC before the start of entecavir treatment, and no evidence of hepatitis C virus or human immunodeficiency virus co-infection and other likely causes of chronic liver disease.

The procedures were performed in accordance with the Helsinki Declaration of 1964 (2013 revision) and approved by the Ethics Committee of Osaka City University Hospital (no. 1646 and 3260). Written informed consent was obtained from each patient.

### Study design

All patients were treated with entecavir for more than 1 year. Entecavir (Baraclude; Bristol-Myers, Tokyo, Japan) was given orally at a dose of 0.5 mg once daily. During the follow-up period, clinical, biochemical, and HBV serological assessments were performed at 1–3-month intervals. Cirrhosis was diagnosed based on a histological examination grade of F4 according to the METAVIR scoring system^44^, using imaging modalities such as ultrasonography, computed tomography (CT), or magnetic resonance imaging (MRI), and based on signs of portal hypertension.

### Patient evaluation

Based on our previous study^8^, we evaluated patients included in this study. The study endpoint was HCC development during entecavir treatment. Patients who developed HCC within 1 year after the start of treatment were excluded. All patients underwent ultrasonography or dynamic CT and/or MRI every 3–6 months for HCC surveillance. HCC was diagnosed based on the presence of arterial hypervascularization and delayed washout on dynamic CT and/or MRI. Patients were followed up until a diagnosis of HCC was confirmed or the last visit before December 2019.

### Laboratory assays

Complete blood counts and serum aspartate aminotransferase (AST), ALT, γ-glutamyltransferase, total bilirubin, and albumin levels were determined using standard procedures. Serum AFP levels were determined via chemiluminescent enzyme immunoassay. Serum concentrations of hyaluronic acid were measured using latex agglutination immunoturbidimetry (Fujirebio Inc., Tokyo, Japan). The serum type IV collagen concentration was measured using latex agglutination turbidimetry (Daiichi Fine Chemical Co., Ltd., Tokyo, Japan). The FIB-4 index was calculated using Sterling’s formula as age (years) × AST (U/L) ÷ platelet count (×10^9^/L) × √ALT (U/L).

HBsAg was measured via chemiluminescent microparticle immunoassay (Architect HBsAg QT; Abbott Japan Corp., Tokyo, Japan). Hepatitis B e antigen (HBeAg) and anti-HBe were detected via chemiluminescent enzyme immunoassay. HBcrAg was also detected via chemiluminescence enzyme immunoassay (Fuji-Rebio, Tokyo). HBV DNA was measured using real-time polymerase chain reaction analysis (COBAS TaqMan HBV Test, ver. 2.0; Roche Diagnostics K.K., Tokyo, Japan). The HBV genotype was determined via enzyme-linked immunosorbent assay using monoclonal antibodies against type-specific epitopes in the preS2-region (Institute of Immunology, Tokyo, Japan). Mutations at nucleotide 1896 in the precore region and nucleotides 1762 and 1764 in the basal core promoter region of HBV DNA were identified via enzyme-linked minisequencing assay (Genome Science Laboratory, Tokyo, Japan). These laboratory assays are similar to those reported in our previous study^8^.

### Soluble immune checkpoint protein assays

Serum samples were collected at baseline. The serum levels of 16 soluble immune checkpoint proteins were measured via multiplexed fluorescent bead-based immunoassay using the Milliplex Map Kit (EMD Millipore Corporation, Danvers, MA, USA) and the Bio-Rad Luminex Bio-Plex- 200 system (Hercules, CA, USA). The 16 soluble proteins of interest were sBTLA, sCD27, sCD28, sTIM-3, sHVEM, sCD40, sGITR, sLAG-3, sTLR-2, sGITRL, sPD-1, sCTLA-4, sCD80, sCD86, sPD-L1, and sICOS. In accordance with the manufacturer’s instructions, 12.5 μL of serum was used for each measurement and all samples were assayed in duplicate; mean values were used for further analysis. For values below the limit of detection, we used 10% of the lowest recorded value as a substitute^19,45^.

In addition, of 13 patients who developed HCC during entecavir treatment, 9 had stored serum samples collected at 6 and 12 months after the start of entecavir treatment, at 6 and 12 months before HCC development, and at the time of HCC development, in which the sPD-1 level was measured via multiplexed fluorescent bead-based immunoassay.

### Statistical analysis

Statistical analyses were performed using JMP software (ver. 12.0; SAS Institute, Cary, NC, USA). Continuous variables were compared using the Mann–Whitney U test, and discontinuous variables using Fisher’s exact test. Receiver-operator curves were generated to obtain the optimal cut-off value for distinguishing between patients with and without HCC. Kaplan–Meier analysis and the log-rank test were used to analyze cumulative rates of HCC development. Cox proportional hazards models were used to identify factors associated with HCC development. Variables with *p*-values < 0.05 in univariate Cox regression analyses were subjected to stepwise multivariate Cox regression analysis. The significance of changes in values between two time points was evaluated using the Wilcoxon signed-rank test. The significance of correlations was evaluated in Spearman’s rank analysis. In two-tailed tests, *p* < 0.05 was taken to indicate statistical significance.

## Supplementary Information

Supplementary Figure 1.

Supplementary Figure 2.

## Abbreviations

AFP: α-Fetoprotein
ALT: Alanine aminotransferase
AST: Aspartate aminotransferase
FIB-4: Fibrosis-4
HBcrAg: Hepatitis B core-related antigen
HBeAg: Hepatitis B e antigen
HBsAg: Hepatitis B surface antigen
HBV: Hepatitis B virus
HCC: Hepatocellular carcinoma
NA: Nucleos(t)ide analogue
sBTLA: Soluble B- and T-lymphocyte attenuator
sCD27: Soluble CD27
sCD28: Soluble CD28
sCD40: Soluble CD40
sCD80: Soluble CD80
sCD86: Soluble CD86
sCTLA-4: Soluble cytotoxic T-lymphocyte-associated antigen 4
sGITR: Soluble glucocorticoid-induced TNFR-related
sGITRL: Soluble glucocorticoid-induced TNFR-related ligand
sHVEM: Soluble herpes virus entry mediator
sICOS: Soluble inducible T-cell co-stimulator
sLAG-3: Soluble lymphocyte-activation gene 3
sTIM-3: Soluble T-cell immunoglobulin and mucin domain-3
sTLR-2: Soluble toll-like receptor 2
sPD-1: Soluble programmed cell death-1
sPD-L1: Soluble programmed cell death-ligand 1
