# Supplementary material for: Soluble programmed cell death-1 predicts hepatocellular carcinoma development during nucleoside analogue treatment
Source: Sci Rep. 2022 Jan 7;12:105. doi: 10.1038/s41598-021-03706-w (PMC8741806; doi:10.1038/s41598-021-03706-w)
Supplement: Supplementary file 1 — Supplementary Figure 1. [file 41598_2021_3706_MOESM1_ESM.pdf]

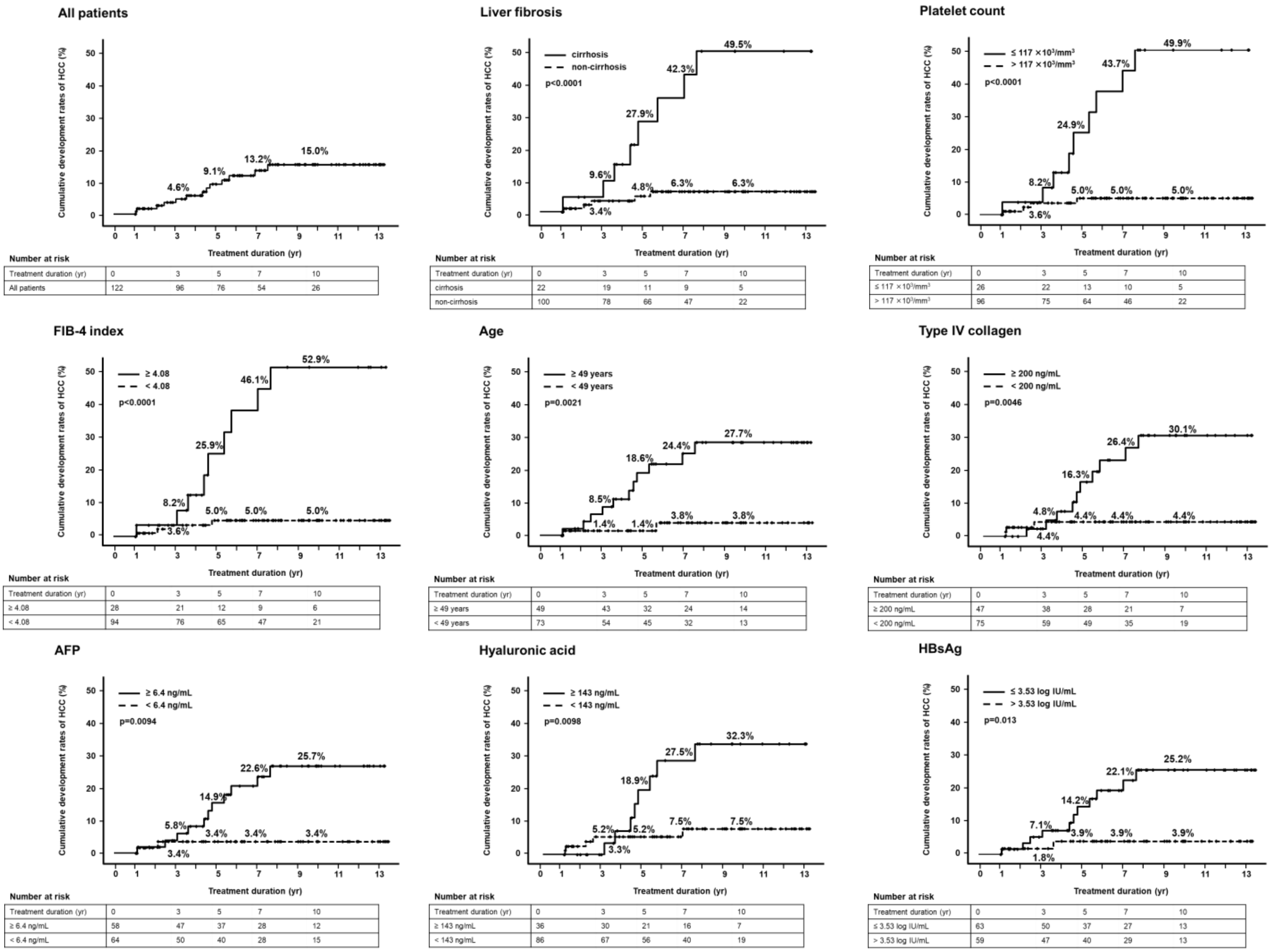

Number at risk

Treatment duration (yr)	0	3	5	7	10
All patients	122	96	76	54	26

Number at risk

Treatment duration (yr)	0	3	5	7	10
cirrhosis	22	19	11	9	5
non-cirrhosis	100	78	66	47	22

Number at risk

Treatment duration (yr)	0	3	5	7	10
≤ 117 ×10³/mm³	26	22	13	10	5
> 117 ×10³/mm³	96	75	64	46	22

Number at risk

Treatment duration (yr)	0	3	5	7	10
≥ 4.08	28	21	12	9	6
< 4.08	94	76	65	47	21

Number at risk

Treatment duration (yr)	0	3	5	7	10
≥ 49 years	49	43	32	24	14
< 49 years	73	54	45	32	13

Number at risk

Treatment duration (yr)	0	3	5	7	10
≥ 200 ng/mL	47	38	28	21	7
< 200 ng/mL	75	59	49	35	19

Number at risk

Treatment duration (yr)	0	3	5	7	10
≥ 6.4 ng/mL	58	47	37	28	12
< 6.4 ng/mL	64	50	40	28	15

Number at risk

Treatment duration (yr)	0	3	5	7	10
≥ 143 ng/mL	36	30	21	16	7
< 143 ng/mL	86	67	56	40	19

Number at risk

Treatment duration (yr)	0	3	5	7	10
≤ 3.53 log IU/mL	63	50	37	27	13
> 3.53 log IU/mL	59	47	40	29	13

Supplementary Figure 1. Cumulative rates of hepatocellular carcinoma (HCC) development in all patients according to the liver fibrosis status, platelet count, fibrosis-4 (FIB-4) index, age, and levels of type IV collagen, α-fetoprotein (AFP), hyaluronic acid, and hepatitis B surface antigen (HBsAg) at baseline.
