# Supplementary material for: Soluble programmed cell death-1 predicts hepatocellular carcinoma development during nucleoside analogue treatment
Source: Sci Rep. 2022 Jan 7;12:105. doi: 10.1038/s41598-021-03706-w (PMC8741806; doi:10.1038/s41598-021-03706-w)
Supplement: Supplementary file 2 — Supplementary Figure 2. [file 41598_2021_3706_MOESM2_ESM.pdf]

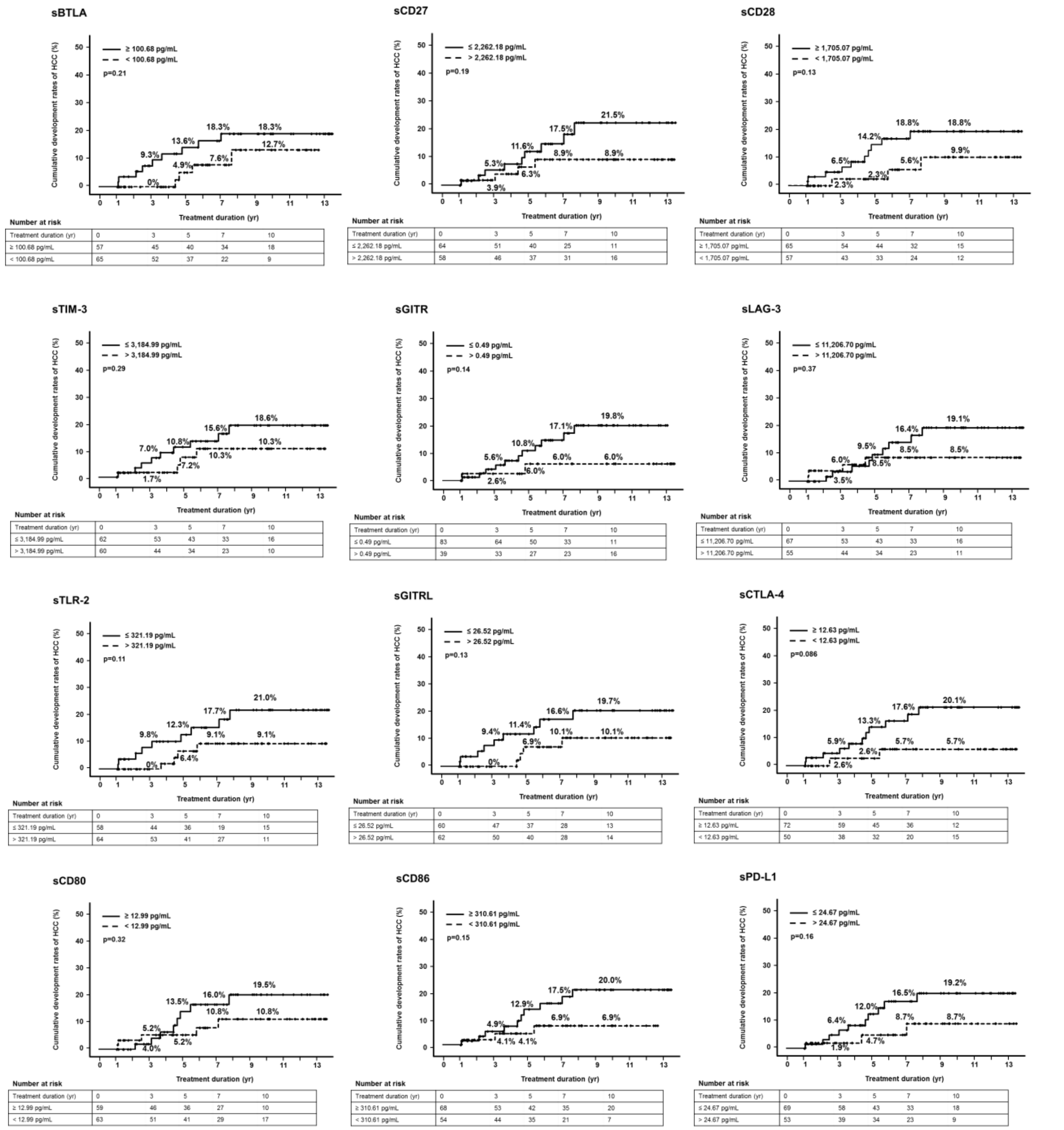

Supplementary Figure 2. Cumulative rates of HCC development according to levels of soluble B- and T-lymphocyte attenuator (sBTLA), soluble (s) CD27, sCD28, soluble T-cell immunoglobulin and mucin domain-3 (sTIM-3), soluble glucocorticoid-induced TNFR-related (sGITR), soluble lymphocyte-activation gene 3 (sLAG-3), soluble toll-like receptor 2 (sTLR-2), soluble glucocorticoid-induced TNFR-related ligand (sGITRL), soluble cytotoxic T-lymphocyte associated antigen 4 (sCTLA-4), sCD80, sCD86, and soluble programmed cell death-ligand 1 (sPD-L1) at baseline.
